# Effect of Leptin on *In Vitro* Nuclear Maturation and Apoptosis of Buffalo (Bubalus bubalis) Oocyte

**Published:** 2014-03-09

**Authors:** Amir Khaki1, Rouzali Batavani, Gholamreza Najafi, Hamid Tahmasbian, Abolfazl Belbasi, Aram Mokarizadeh

**Affiliations:** 1 Department of Clinical Sciences, Division of Theriogenology, Faculty of Veterinary Medicine, Urmia University, Urmia, Iran; 2 Department of Basic Sciences, Division of Embryology, Faculty of Veterinary Medicine, Urmia University, Urmia, Iran; 3 Department of Immunology, Faculty of Medicine, Kurdistan University of Medical Sciences, Sanandaj, Irann

**Keywords:** Buffalo, Oocyte, Leptin, *In Vitro* Maturation, Apoptosis

## Abstract

**Background:**

Leptin, as a 16 kDa adipokine, is a pleiotropic cytokine-like hormone that primarily secreted from adipose tissue. It also involves in the regulation of energy homeostasis, neuroendocrine function, immunity, lipid and glucose homeostasis, fatty acid oxidation, angiogenesis, puberty and reproduction. The aim of this study was to investigate the effects of *in vitro* addition of leptin to *in vitro* maturation (IVM) medium on buffalo oocyte maturation and apoptosis.

**Materials and Methods:**

In this experimental study, Ovaries from apparently normal reproductive organs of slaughtered adult buffaloes (Bubalus bubalis) with unknown breeding history were collected from Urmia Abattoir, Urmia, Iran, and were transported immediately to the laboratory in a thermos flask containing sterile normal saline with added antibiotics. Oocytes were aspirated from 2-8 mm visible follicles of the ovaries using an 18-G needle attached to a 10 ml syringe. IVM medium included tissue culture medium-199 (TCM-199), 10% fetal bovine serum (FBS), 22 µg/ml sodium pyruvate, 0.5 IU/ml ovine follicle-stimulating hormone (oFSH), 0.5 IU/ml ovine luteinizing hormone (oLH), 1 μg/ml oestradiol, 50 μg/ml gentamycin, and leptin [0 (control), 10, 50, and 100 ng/ml]. The good quality buffalo oocytes (batches of 10 oocytes) were placed in a culture plate containing six 50 μl droplets of maturation medium, covered with sterilized mineral oil, and then incubated at 38.5˚C with 5% CO_2_ in air for 24 hours. The maturation of oocytes was evaluated under a stereomicroscope by detecting the first polar body extrusion of oocytes. FITC-Annexin V propidium iodide (PI) staining method was used to detect oocyte apoptosis.

**Results:**

From a total of 115 collected ovaries, 1100 oocytes were recovered among which 283 oocyte were suitable for IVM. In the groups of leptin treated with 0 (control), 10, 50 and 100 ng/ml, the percentage of oocytes maturation was 74.65, 83.81, 77.85, and 75.40%, while the percentage of oocytes apoptosis was 9.83, 9.54, 9.93, and 10.42%, respectively. Our results showed that addition of 10 ng/ml leptin to buffalo IVM medium increased oocyte maturation, significantly, as compared with that in control group. However, addition of leptin to IVM medium had no significant influence on buffalo oocyte apoptosis. Conclusion: Our findings suggested that addition of 10 ng/ml leptin to IVM medium of buffalo oocyte can improve oocyte nuclear maturation. Furthermore, we showed that there is no relation between *in vitro* addition of leptin to buffalo oocyte IVM medium and oocyte apoptos

**Conclusion:**

Our findings suggested that addition of 10 ng/ml leptin to IVM medium of buffalo oocyte can improve oocyte nuclear maturation. Furthermore, we showed that there is no relation between *in vitro* addition of leptin to buffalo oocyte IVM medium and oocyte apoptosis.

## Introduction

Production of calves with high genetic paternity is an increasingly important area for *in vitro* embryo production (IVEP) of buffalo. In comparison with cattle, reproductive technologies have poorly developed for buffalo. This may be due to reproductive physiology characteristics such as late maturity, silent oestrus, distinct seasonal reproductive pattern and long calving interval in this species (1, 2). To consider the low oocyte maturation rate (69.5-72.3%) (2), poor oocyte recovery rate, lack of standardization for technical factors in the IVEP and low *in vitro* fertilization (IVF) performance of buffalo bull spermatozoa (3, 4), we determined to study further about this merit mammal. 

Leptin is defined as a 16 kDa adipokine, primarily secreted by adipose tissue, and a multifunctional hormone (5). Major role of leptin in control of reproductive function is now firmly established. The ob/ob mice (lacking functional leptin with mutation in leptin gene) are infertile. Fertility of both female and male ob/ob mice is restored by leptin administration (6, 7).

Leptin is expressed in murine (8, 9), human (10), porcine (11, 12), bovine (13), and equine (14) oocytes. However, in some animals, mRNA transcript has not identified in the oocyte (9, 15, 16); therefore, some scientists have suggested that it may be produced elsewhere and transported into the oocyte (11). Leptin receptor has been detected in granulosa cells, cumulus cells and oocytes in human (10, 17, 18), mouse (8, 9, 15), rat (19), rabbit (20), porcine (11, 12, 21), ewe (22) and bovine (23, 24). Also, leptin and its receptor were shown to be present in bovine corpus luteum (25). The presence of leptin receptor in oocyte and embryo [bovine (26), porcine (27), and rabbit (28)] has been suggested that both oocytes and preimplantation embryos could react to leptin.

There are evidences that addition of leptin into IVM medium can stimulate oocyte maturation in porcine (11, 29, 30), murine (9), bovine (23, 24, 26, 31), rabbit (32-34), and equine (14). It seems that leptin enhances oocyte maturation by mitogen activated protein kinase (MAPK) pathway phosphorylation (11). There are contradictory reports about the effect of added leptin to *in vitro* culture (IVC) medium for improving embryo development. Some studies have supported its benefits (12, 16, 26), while some others have reported no effect of leptin (35, 36).

Researches in and around apoptosis, the programmed cell death, have increased substantially since the early 1990s. It has been proved that treatment with exogenous leptin in ob/ob mice and human with congenital defect in leptin producing can reintegrate the immune response (37-39) and can reduce thymus atrophy with an increase in cellularity (38). Leptin administration in rat reduced incidence of oocyte apoptosis in vivo (40). Furthermore, it was demonstrated that in leptin deficient mice, folliculogenesis is impaired and the apoptosis of granulosa cells is increased (41).

With our knowledge, there is no report about the effect of *in vitro* addition of leptin to IVM medium on buffalo oocyte maturation and apoptosis. With this background, the aim of this study was to investigate the effects of *in vitro* addition of leptin to IVM medium on buffalo oocyte maturation and apoptosis. 

## Materials and Methods

### Chemicals and supplies

All chemicals and reagents were purchased from Sigma Chemical Co., St. Louis, Mo, USA, unless otherwise stated. Plastic dishes and six-well plates were obtained from Petes Co., USA.

### Collection and processing of ovaries

In this experimental study, ovaries from apparently normal reproductive organs of adult buffaloes (Bubalus bubalis) of unknown breeding history slaughtered in Urmia Abattoir, Urmia, Iran (37˚ 33΄ N, 45˚ 4΄ E) were collected within 10 minutes after slaughter and transported to the laboratory in a thermos flask (32- 33˚C) containing sterile normal saline supplemented with antibiotics (1000 IU/ml penicillin G and 1 mg/ml streptomycin) within 1 hour of slaughter (42). In the laboratory, extraneous tissue was removed and ovaries were washed thoroughly for four times in normal saline supplemented with 50 µg/ml gentamycin. Precautions were taken to minimize bacterial contamination by conducting procedures in highly sterile conditions (43). 

### Recovery of oocytes

**Fig 1:**
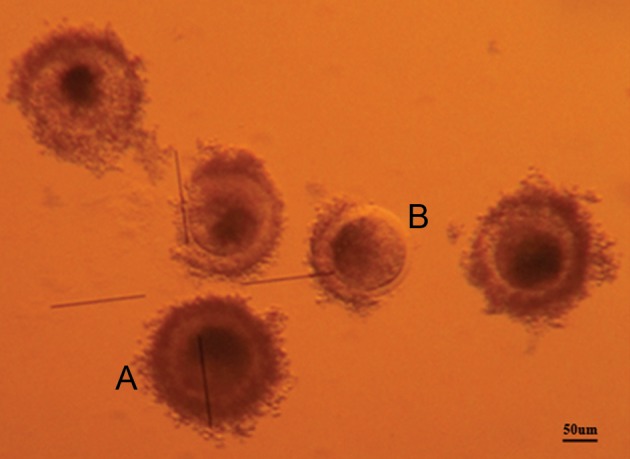
Recovered buffalo oocytes; good quality oocyte for IVM (magnification ×125) (A); poor quality oocyte for IVM (B).

Due to remaining of some follicles embedded in the ovary, aspiration of oocytes from the buffalo ovaries is considered as a big challenge, so, in the first step, oocytes were aspirated from 2-8 mm visible follicles of the ovaries using an 18-G hypodermic needle attached to a 10 ml disposable plastic syringe containing aspiration medium [TCM-199 fortified with 10% fetal bovine serum (FBS; Invitrogen, USA)]. In the second step, the ovaries were dissected and washed with aspiration medium to recover the remaining oocytes. The aspirated fluid was transferred to the 37˚C pre-warmed petridish. Cumulus oocyte complexes were isolated under a low-power magnification zoom stereo microscope (Nikon, Japan). For assessment of oocytes quality, the classification of Yadav et al. (44) was used, and oocytes were graded by morphological appearance of the cumulus cells investments and homogeneity of ooplasm under a zoom stereomicroscope (×110) as following (Fig 1): i. A grade: cumulus oocyte complex (COC) with 4 or more layers of compact cumulus cells surrounding the zona pellucida with evenly granulated cytoplasm, ii. B grade: COC with 1-3 layers of compact cu- mulus cells surrounding the zona pellucida with evenly granulated cytoplasm, iii. C grade: oocyte with fibrous (expanded) cumulus layers surrounding the zona pellucida, and iv. D grade: oocyte without cumulus cells and an irregular ooplasm. Only grades A and B oocytes were employed for *in vitro* maturation (IVM).

The collected oocytes were washed two time in fresh pre-warmed 4-(2-hydroxyethyl)-1-piperazineethanesulfonic acid (HEPES) buffered Tyrode’s medium (TL-HEPES) followed by two washings in culture medium containing TCM-199 supplemented with 10% FBS, and were then subjected to a final wash with IVM medium before transferring to the drops (45).

This study was performed between April and June (2012), two times in a week.

### IVM

**Fig 2:**
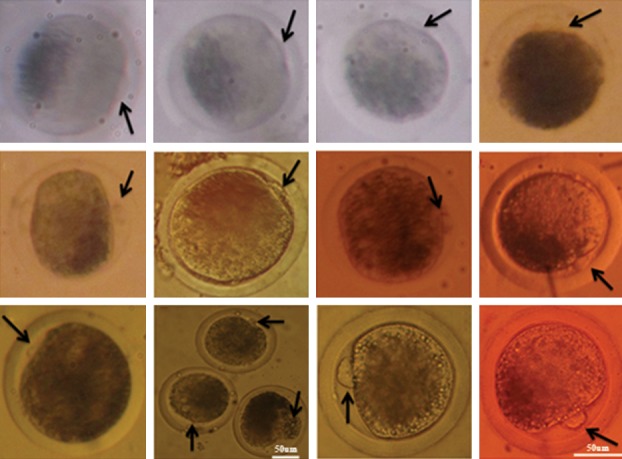
Mature buffalo oocytes; arrows show polar body indi- cating buffalo oocyte maturation (magnification All-10×200, 10 ×100).

In vitro maturation medium included TCM-199, 10% FBS, 22 µg/ml sodium pyruvate, 0.5 IU/ml ovine follicle-stimulating hormone (oFSH), 0.5 IU/ml ovine luteinizing hormone (oLH), 1 μg/ ml oestradiol, 50 μg/ml gentamycin, and leptin (mouse recombinant leptin) [0 (control), 10, 50, and 100 ng/ml] (45, 46). Good quality buffalo oocytes (batches of 10 oocytes) were placed in a culture plate containing six droplets of 50 μl of maturation medium, covered with sterilized mineral oil, and then incubated at 38.5˚C with 5% CO_2_ in air for 24 hours. Oocytes maturation was evaluated under a stereomicroscope by detecting the first polar body extrusion which is the indicator of oocyte attaining the metaphase II stage (44) (Fig 2).

### Apoptosis detection

Fluorescein isothiocyanate-Annexin V/propidium iodide (FITC-Annexin V/PI) double staining method was used to detect apoptosis (47). Specific binding of FITC-annexin V along with staining with PI was performed with an apoptosis detection kit (BD Pharmingen™-556570, USA) according to the manufacturer’s instructions. Briefly, after 24-28 hours incubation of oocytes in 5% CO_2_ incubator, 10 oocytes were washed one time with TCM199. Then, oocytes were diluted in 200 µl ABB buffer and were located gently on the siliconized slides. Afterward, 10 µl Annexin–V was added to them. The samples were incubated at room temperature in the dark for 20 minutes. Then, 1µg/ml PI was added to the samples and apoptotic oocytes were immediately detected under a fluorescence microscope (Nikon Co., Japan).

**Fig 3:**
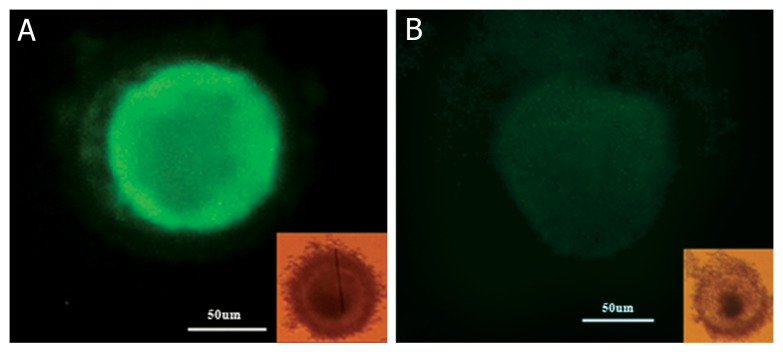
Annexin–V staining for detecting oocyte apoptosis (magnification ×250); apoptotic oocyte (A), non-apoptotic oocyte (B).

In Annexin-V staining, the membranes containing phosphatidylinositol binded to fluorescent dye due to inversion of oocytes membrane and apoptosis, so under the fluorescence microscope is detectable as a green staining. Non apoptotic oocytes are not stained (Fig 3).

### Statistical analysis

Data on maturation and apoptosis were analyzed using software package used for statistical analysis (SPSS) (Version 19; SPSS Inc., Chicago, IL, USA). Statistical mean and standard error of mean (SEM) were calculated for each group and were compared by one-way analysis of variance (ANOVA). Duncan’s test was used for the multiple comparison and least significant difference (LSD) values were calculated for significant difference between control group and treatment groups. Differences were considered significant when p≤0.05. 

## Results

Out of the 1100 oocytes recovered from a total of 115 collected ovaries, 238 were suitable for IVM (Table 1). 

**Table 1:** The number of used ovaries, recovered oocytes, and good quality oocytes for IVM, while showing apoptosis in different leptin treated groups


Leptin concentration ng/ml	Number of ovaries	Recovered oocytes	Good quality oocyte

**0**	29	277	72
**10**	29	276	71
**50**	29	274	70
**100**	28	273	70


### Effect of leptin on IVM

**Fig 4:**
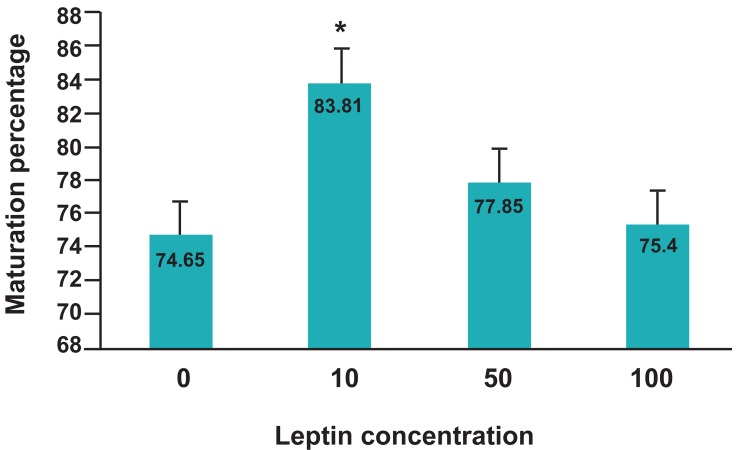
Effect of different leptin concentrations on oocyte maturation. There is a significant difference (p<0.05)

The percentage of oocyte maturation in control group and leptin treated groups is mentioned in figure 4. Addition of 10 ng/ml leptin to buffalo IVM medium increased oocyte maturation, significantly (p<0.05). 

### Effect of leptin on oocyte apoptosis

**Fig 5:**
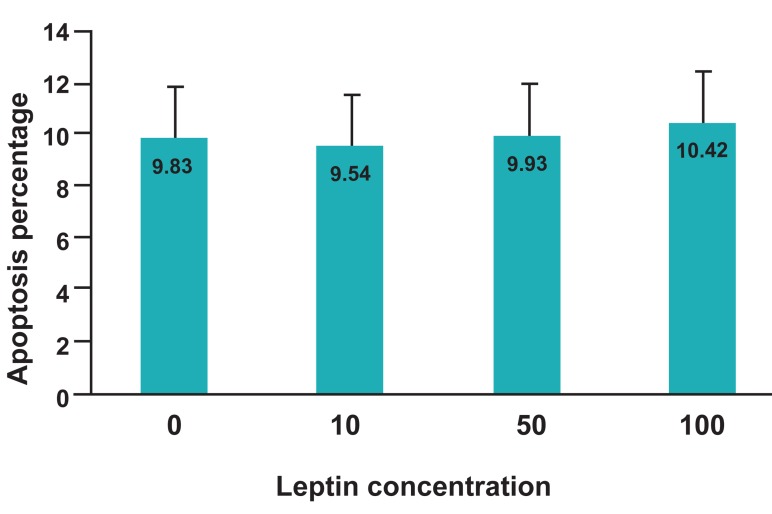
Effect of different leptin concentrations on oocyte apoptosis.

 The percentage of oocyte apoptosis in control group and leptin treated groups is mentioned in fig 5. There was no significant difference in buffalo oocytes apoptosis between control group and the other leptin treated groups (p>0.05).

## Discussion

The present study was carried out to investigate the effects of different concentrations of leptin added during the *in vitro* maturation of buffalo oocytes on percentage of mature and apoptotic oocytes. It has been established that the addition of leptin at physiological concentrations (~ 10 ng/ ml) enhances ability of *in vitro* maturation of adult bovine oocytes (23, 26, 31). Craig et al. (11) and Kun et al. (30) observed that 10 ng/ml leptin during pig oocytes maturation caused higher oocyte maturation rates, significantly. Moreover, in horses, Lange Consiglio et al. (14) demonstrated that the addition of leptin in the range between 10 and 1000 ng/ml increases the maturation rate of equine oocytes, although the statistical significance was observed only at the concentration of 100 ng/ml. Moreover, Arias-Alvarez et al. (32) showed that addition of leptin to IVM medium at physiological dose (10 ng/ml) improves both meiotic and cytoplasmic maturation of rabbit oocytes, whereas an excessive leptin concentration does not have the extra beneficial effect. These results are in line with our observation in buffalo which demon- strated, for the first time, that addition of 10 ng/ ml leptin to buffalo oocyte IVM medium improves oocyte maturation. Lu et al. (48), indeed, studied the effect of leptin on *in vitro* development of buffalo embryos, showing that supplementation of 10 and 100 ng/ml leptin to *in vitro* culture (IVC) medium of buffalo embryos could enhance blastocyst development in buffalo. The optimal concentration of leptin in their procedures was 10 ng/ml and they did not add leptin to IVM medium .

 Apoptosis has an important role in mammalian development as a quality control mechanism for eradicating damaged, non-functional, and abnormal cells, as well as those cells that are in incorrect place (49, 50). It has been shown that "leptin protects mice from starvation-induced lymphoid atrophy and increases thymic cellularity in ob/ob mice" (38). There are studies reported leptin exerts anti-apoptotic activity in T cells (51), monocytes (52), neuroblastoma cells (53), neutrophils (54), hippocampal neurons (55) and murine dendritic cells (56), while inducing apoptosis in human bone marrow stromal cells (57).

The reason for these opposing responses in different cell types is unknown, but differences in the expression patterns of leptin receptors and as- sociated signaling molecules may play an important role (58). Beneficial effect of leptin on oocyte maturation proposes a role for leptin as a survival factor which minimizes cell damage. Therefore, we investigated the effect of leptin on oocyte apoptosis after IVM.

Our finding showed that leptin had no significant effect on oocyte apoptosis after IVM in comparison with that in control group. But, there is an in vivo study which reported that leptin administration in rats can rescue oocytes and follicles from atresia by attenuation of apoptosis (40). Furthermore, leptin deficiency in mice is associated with suppression of ovarian folliculogenesis and with an increase in ovarian granulosa cell apoptosis (41).

Ikeda and co-workers reported that an increase in the extent of apoptosis may alter connectivity between the cells of the cumulus-oocyte, and subsequently reduces the quality of oocytes, while the degree of apoptosis has also negative correlation with the developmental competence of bovine cumulus-oocyte complexes (59). Leptin supplementation during bovine oocyte maturation reduces the proportion of terminal deoxynucleotidyl transferase dUTP nick end labeling (TUNEL)-positive cells per blastocyst (26). Furthermore, it has been shown that physiological doses of leptin during maturation of oocyte cumulus complex increase expression of baculoviral inhibitor of apoptosis protein repeat-containing 4 (BIRC4) mRNA transcripts, while decrease the cumulus cells apoptosis and show no beneficial effect on bovine oocyte maturation (60). Furthermore, Paula-Lopes et al. (31) which studied the *in vitro* effect of leptin on nuclear maturation of bovine oocyte reported that leptin reduces apoptosis of cumulus cells, but have no effect on oocyte apoptosis. Similarly, Jin et al. (61) showed that addition of leptin during IVM of porcine oocytes had no effect on apoptotic cells in blastocysts. It has been demonstrated that leptin has no effect on expression of apoptotic genes in bovine blastocyst *in vitro* (28). Furthermore, Cordova and co-workers acclaimed that leptin not only has no effect on oocyte apoptosis, but also high leptin concentration increases oocyte apoptosis during IVM of prepubertal calf oocytes (62). With our knowledge, there is no report about the effect of leptin on apoptosis of buffalo oocyte. Regarding to our finding and the other reports, we can conclude that leptin has no effect on oocyte apoptosis *in vitro*. 

## Conclusion

Our findings showed that addition of 10 ng/ml leptin to IVM medium of buffalo oocytes can increase oocyte nuclear maturation, and we recommend adding this hormone to IVM medium for improving oocyte maturation of this merit mammal. Also, our study showed that leptin has no effect on buffalo oocyte apoptosis after IVM. 
